# Reply to Gambazza et al. Cystic Fibrosis, New Frontier: Exploring the Functional Connectivity of the Brain Default Mode Network. Comment on “Elce et al. Impact of Physical Activity on Cognitive Functions: A New Field for Research and Management of Cystic Fibrosis. *Diagnostics* 2020, *10*, 489”

**DOI:** 10.3390/diagnostics11061002

**Published:** 2021-05-31

**Authors:** Ausilia Elce, Valentina Elce, Alessandro Del Pizzo

**Affiliations:** 1 CEINGE Biotecnologie Avanzate SCarl, Via Gaetano Salvatore 486, 80145 Napoli, Italy; 2 Dipartimento di Scienze Umanistiche, Università Telematica Pegaso, Via Porzio, Centro Direzionale, isola F2, 80143 Napoli, Italy; 3 MoMiLab, IMT School for Advanced Studies, Piazza San Francesco 19, 55100 Lucca, Italy; valentina.elce@imtlucca.it; 4 Dipartimento di Fisica, University of Pisa, Largo Bruno Pontecorvo, 3, 56127 Pisa, Italy; aledelpizzo@gmail.com

We appreciate the interest in our review and we are grateful for the comment by Gambazza S. et al., because it investigates new aspects of functional connectivity in CF patients, applying fMRI to resting state study for neuropsychological assessment in trained CF subjects.

The preliminary results seem to be very encouraging and require further research to assess the specific exercise-induced modifications of CF patient’s cognitive functions. The topic falls in the new field of research for CF management proposed by our review. A progression in this field may be a turning point for the approach to Cystic Fibrosis patients from the earliest interventions. 

In the last year, a limited number of studies focusing on CF neurocognitive aspects were published. In 2020, Gold A et al. observed improvements in verbal learning, aspects of mood, behaviour, and adaptive functioning in a group of children (CF and non-CF) undergoing lung transplantation, although a weakness of executive functions was also observed after transplantation [1]. These data suggest that further studies are required to investigate specific CF cerebral connectivity and that educational and neuropsychological strategies should be adopted to prevent the cognitive alterations observed in CF patients.
